# Forages and pastures symposium: an update on in vitro and in situ experimental techniques for approximation of ruminal fiber degradation

**DOI:** 10.1093/jas/skad097

**Published:** 2023-07-04

**Authors:** Jamie L Foster, William B Smith, F Monte Rouquette, Luis O Tedeschi

**Affiliations:** Texas A&M AgriLife Research, Beeville, TX, USA; Department of Animal Sciences, Auburn University, Auburn, AL, USA; Texas A&M AgriLife Research, Overton, TX, USA; Department of Animal Science, Texas A&M University, College Station, TX, USA

**Keywords:** digestion kinetics, fiber degradation, indigestible fiber, in situ, in vitro, ruminant nutrition

## Abstract

Static quantification measures of chemical components are commonly used to make certain assumptions about forage or feed nutritive value and quality. In order for modern nutrient requirement models to estimate intake and digestibility more accurately, kinetic measures of ruminal fiber degradation are necessary. Compared to in vivo experiments, in vitro (**IV**) and in situ (**IS**) experimental techniques are relatively simple and inexpensive methods to determine the extent and rate of ruminal fiber degradation. This paper summarizes limitations of these techniques and statistical analyses of the resulting data, highlights key updates to these techniques in the last 30 yr, and presents opportunities for further improvements to these techniques regarding ruminal fiber degradation. The principle biological component of these techniques, ruminal fluid, is still highly variable because it is influenced by ruminally fistulated animal diet type and timing of feeding, and in the case of the IV technique by collection and transport procedures. Commercialization has contributed to the standardization, mechanization, and automation of the IV true digestibility technique, for example, the well-known Daisy^II^ Incubator. There has been limited commercialization of supplies for the IS technique and several review papers focused on standardization in the last 30 yr; however, the IS experimental technique is not standardized and there remains variation within and among laboratories. Regardless of improved precision resulting from enhancements of these techniques, the accuracy and precision of determining the indigestible fraction are fundamental to modeling digestion kinetics and the use of these estimates in more complex dynamic nutritional modeling. Opportunities for focused research and development are additional commercialization and standardization, methods to improve the precision and accuracy of indigestible fiber fraction, data science applications, and statistical analyses of results, especially for IS data. In situ data is typically fitted to one of a few first-order kinetic models, and parameters are estimated without determining if the selected model has the best fit. Animal experimentation will be fundamental to the future of ruminant nutrition and IV and IS techniques will remain vital to bring together nutritive value with forage quality. It is feasible and important to focus efforts on improving the precision and accuracy of IV and IS results.

## Teaser

The in vitro and in situ techniques are as important to ruminant nutrition as they were 30 yr ago. There have been improvements in methodology and mathematical modeling of results, but there is still tremendous opportunity to improve the precision and application of in vitro and in situ techniques.

## Introduction

In 1991, the International Symposium on Forage Cell Wall Structure and Digestibility was held at the U.S. Dairy Forage Research Center in Madison, WI, United States, and culminated in the seminal publication of *Forage Cell Wall ­Structure and Digestibility* (Jung et al., 1993). This conference represented a multidisciplinary congregation of scientists with the primary intent of developing a comprehensive compendium on the state of knowledge of the forage cell wall. From the perspective of the ruminant nutritionist, significant interest was generated in the experimental measurement of disappearance as it appeared that the cell wall was the primary limiting factor to forage digestibility (Jung and Allen, 1995). In this light, the in vitro (**IV**) and in situ (**IS**) techniques are mentioned throughout the publication, primarily in the chapter written by Mertens (1993). The IV technique removes ruminal microbes from the animal for incubation of substrate in a separate vessel, while the IS technique relies on suspending the substrate of interest in the rumen to use natural biological interactions. This review focuses on IV dry matter digestibility (**IVDMD**) and IV organic matter digestibility two-stage incubation technique with microbial incubation followed by pepsin as presented by Tilley and Terry (1963) and techniques that evolved from IVDMD, such as IV true digestibility (**IVTD**) which uses extraction with neutral detergent fiber (**NDF**) solution (Van Soest et al., 1966). Time-series or IV gas production (**IVGP**) techniques are excluded from this review as they are addressed by Tedeschi et al. (2023). The IS technique uses a vessel that is not degraded in the rumen that holds substrate within the rumen for incubation. The IS procedure was first described for the study of ruminal digestive kinetics by Quin et al. (1938). There are numerous references for the IS technique methodology and the impact of bag characteristics, sample characteristics, dietary effects, analytical techniques, and correction for microbial contamination on the accuracy and precision of resulting data (ARC, 1984; Nocek, 1988; Michalet-Doreau and Ould-Bah, 1992; Vanzant et al., 1998). Both techniques result in measures of substrate or sample disappearance measures after incubation, termed “digestibility” or “degradation.” The focus of this review is the application of the IV and IS technique to ruminal fiber degradation although they have obvious importance to the study of feed and fiber constituents other than fiber, including crude protein.

The IV and IS techniques are important in ruminant animal nutrition because they bridge measures of forage quality and nutritive value. Forage quality is a measure of animal gain or milk production when forage is not limiting, whereas nutritive value is measured by laboratory methods that are disconnected from the biological component of the animal consuming the forage. In vitro dry matter digestibility and IVTD are correlated to in vivo digestibility and intake (Barnes and Marten, 1979; Van Soest, 1994). Application of IV results to ruminant performance is an extrapolation of results that requires mathematical modeling and specific assumptions about the digesta composition and kinetics through the different compartments in the gastrointestinal tract of the ruminant animal (Vinyard and Faciola, 2022; Tedeschi et al., 2023). Though the ruminant species for which predictive modeling is applied should be the same species of ruminal fluid collection or incubation (Foster et al., 2007). Accurate digestion of kinetic data is crucial for incorporation into dynamic models (Weiss, 1994). With modeling, IV and IS techniques can bring together measures of fiber, which are laboratory measures, to in vivo apparent digestibility and intake, which are animal-influenced measures, as a proxy to expensive and time- and labor-consuming feeding trials, which require much larger amounts of feedstuffs and more animal experimental units. Ranking of IV or IS results can help determine forages in breeding programs that are most suitable for advancement (Foster et al., 2007), impact of genetic traits on potential animal performance (McCuistion et al., 2017), the ideal maturity stage for harvest (Foster et al., 2012), harvested forage preservation method (Foster et al., 2011, 2012), harvested forage preservation aids such as enzyme or microbial applications before ensiling (Thomas et al., 2013, 2014; McCuistion et al., 2017; Foster et al., 2019), alternative uses of row crop residues (Foster et al., 2012, 2019), supplementation regimes (Smith et al., 2017), and among many other applications.

There has been progressing since the thorough assessment and summary of the state of the knowledge of IV and IS techniques presented by Mertens (1993). The following year, on the 25th anniversary of the first National Conference on Forage Quality, Evaluation, and Utilization, Weiss (1994) reviewed estimating forage digestibility by laboratory methods including factors that affect and control accuracy, precision, and reproductivity of estimates of digestibility and compared results from various methods of estimating forage digestibility. There have been exhaustive reviews of both IV (Weiss, 1994; López, 2005; Tassone et al., 2020; Vinyard and Faciola, 2022) and IS (Nocek, 1988; Vanzant et al., 1998; López, 2005) methods of digestibility estimation. This review presents the historical context of the state of knowledge at the time of the 1991 symposium, summarizes key advances in the IV and IS techniques over the last 30 yr, and discusses opportunities for future directions for these vital assays.

## Historical Context

### Limitations of in vitro and in situ methodology

Mertens (1993) outlined the limitations of both the IV and IS methods for determining the digestibility of forage cell walls. The limitation of using the IV system for evaluating feedstuff disappearance is its removal from the in vivo fermentation environment (Mertens, 1993). Control of the incubator that results in a consistent fermentation environment favors the IV technique. Unfortunately, most IV methods for measuring substrate disappearance at 48-h, such as IVDMD, IVOMD, and IVTD, are not appropriate for use in measuring digestive kinetics due to suboptimal fermentation conditions and reduced degradation at the earlier time points (Mertens, 1993).

Despite supporting viewpoints about the validity of IV techniques for estimating fiber digestibility (Krizsan et al., 2013), the IV method cannot combine the effects of diet and animals for measuring digestion kinetics. The IS technique combines the effects of diet and animal and thus poses complications including that the ruminal environment, unlike the tube or flask, is not in a steady state, and feeding time and frequency impact results (Mertens, 1993). Second, the IS procedure has always required porous bags that allow the inflow and outflow of microbial cells and particulate matter, thereby complicating the measurement of disappearance (Mertens, 1993). Van Hellen and Ellis (1977) recommended using incubation bags with porosity of no greater than 10 µm after finding that porosities of 1.2 µm restricted the efflux of digested particles while porosities of 75 or 100 µm allowed for excessive influx of ruminal fiber particles and outflux of the substrate.

### Mathematical modeling of digestive kinetics

**Figure 1.**
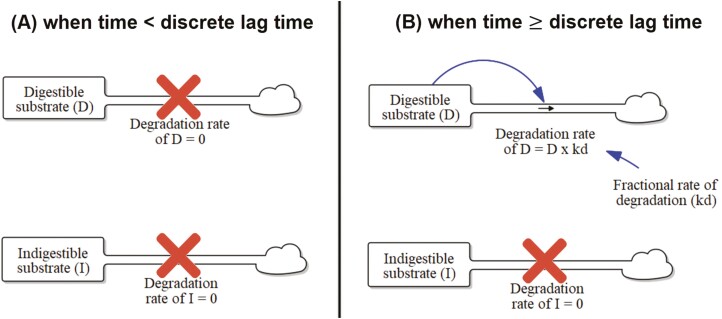
Visual representation of first-order kinetics model with two pools (digestible and indigestible) and the fractional rates of degradation (k_d_) of the digestible pool for when fermentation time is before (A) or after (B) the discrete lag time. The indigestible pool has no degradation at all times.

From a mathematical modeling perspective, incorrect data, erroneous mathematical models, and improper data fitting to the mathematical model are the three primary sources of error in estimating digestion kinetics (Mertens, 1993, 2005). The technique of IV or IS must not limit degradation by microbes, or the intrinsic amount and rate of digestion of the incubated feed or forage cannot be measured. Mertens (1993) also summarized the concept that the use of indigestible neutral detergent fiber (**iNDF**) determined from 72-h IV incubation to calculate potentially degradable NDF (**pdNDF**) was necessary to improve modeling efforts of digestion kinetics as previously described (Waldo et al., 1969; Smith et al., 1971, 1972; Mertens, 1993), though recent analyses have shown that even after 288-h IS incubation, pdNDF still exist for forages containing condensed tannins (Norris et al., 2019). As shown in Figure 1, regressions of pdNDF and time were determined to follow first-order digestion kinetics (Smith et al., 1971, 1972; Mertens, 1993, 2005).

Based on the works of Waldo et al. (1972) and Mertens (1973), Equation (1) is the most common first-order model with a lag time to statistically analyze the digestion kinetics of fiber degradation data (Mertens and Lofton, 1980).


$$\begin{array}{l} R_{\left(t\right)}=\left\{ \begin{array}{l} B+C,\;t<L\\ \;B\times \left(e^{-kd(t-L)}\right)+C,\;t\;\geq L \end{array}\right. \end{array}$$ (1)

where *R*_(*t*)_ is the total indigestible residue at any time incubated in the rumen *t*, *B* is the insoluble potentially degradable fraction, *k*_*d*_ is the rate of digestion of the potentially degradable fraction, *L* is the discrete lag time prior to degradation, and *C* is the fraction not digested.

**Figure 2.**
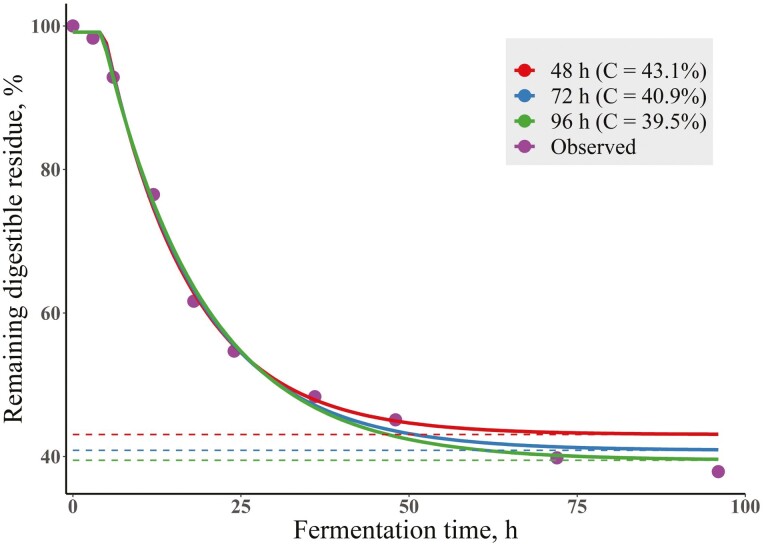
The remaining digestible residue of guinea grass (*Panicum maximum*) forage (purple circles) and the fitting of the exponential model with lag using three different end points (48, 72, and 96 h) of fermentation. The parameters for Equation (1) were B = 154.7 g, C = 118.8 g, kd = 0.078 h^-1^, and L = 4.63 h for 48 h (red line), B = 160.8 g, C = 112.7 g, kd = 0.071 h^-1^, and L = 4.49 h for 72 h (blue line), and B = 164.6 g, C = 108.9 g, kd = 0.066 h^-1^, and L = 4.29 h for 96 h (green line). The horizontal, dashed lines represent the C as a percent of the amount incubated (275.8 g). Adapted from Mertens (1973).

Indigestible fiber is a crucial measure since this value is the basis for the determination of the amount of degradable fiber and the subsequent calculation of the rate of degradation (Mertens, 1973, 1993). It is important that measures of indigestible fiber are measures of the substrate, which may require incubation for a more extended time period, perhaps even longer than 288 h for some feedstuffs, than the digesta would generally remain in the rumen. The estimation of indigestible fiber can be dependent upon the end point of incubation, and this influences the fit of the statistical modeling of degradation kinetics (Mertens, 1973), as shown in Figure 2. There is exhaustive literature on the limitations of estimating digestion kinetics with IV and IS techniques: for example, the rate of mixing, comminution, and passage cannot be calculated from these values and is usually not incorporated explicitly in the analyses (Vieira et al., 2008a, 2008b), but instead combined into one single rate by assuming an aging chain structure (Tedeschi and Fox, 2020a, 2020b). Nevertheless, safeguarding the limitations and simplifications, these techniques have value to ruminant nutrition. Ellis et al. (1994) stated that “the nutritive value of a feedstuff is determined by its content of chemical entities and their transformations to nutrients required by the animal”. Digestive transformations are determined by the intrinsic attributes of the forage and by their interactions with the kinetic processes of digestion. Quantitative expressions of the kinetics of digestion and passage are needed to more precisely estimate the quantity and composition of nutrients digested from forages and their subsequent efficiency of utilization by the animal.”

## 30 Yr Later: What Progress Has Been Made Since 1993?

### Developments in the in vitro technique

Many developments designed to enhance and fine-tune the IV procedure have revolved around the technique’s standardization, mechanization, and automation. Likewise, there have been numerous instances in which researchers have noted ethical questions or shortages of labor needed for the maintenance of ruminally fistulated animals (Mansfield et al., 1995; Castillo and Hernández, 2021; Deitmers et al., 2022), thus necessitating a benchtop technique suitable for feedstuff evaluation. The IVGP technique is a batch culture technique that assesses gas production throughout the incubation period using pressure sensors attached to a needle inserted in the flasks. The IVGP can be closed (i.e., no gas is released) or open (i.e., gas is released at pre-determined thresholds). Besides obtaining the IV digestibility of the incubated substrate, IVGP also provides data to calculate the fermentation dynamics. Tedeschi et al. (2023) provide additional information about IVGP.

#### Batch culture

Until 1991, the IV digestibility system relied heavily on substrates being separated into individually inoculated vessels (Tilley and Terry, 1963; Goering and Van Soest, 1970). This technique was labor and material intensive, with users often constrained by available space to incubate in tubes or flasks. In 1995, a batch culture technique was introduced whereby samples could be exposed to a common rumen inoculum in a single, large container for comparative measures of dry matter (**DM**) and fiber disappearance (Traxler et al., 1995). This method was based on the IVTD method with a neutral detergent solution wash after incubation and was further evaluated by Vogel et al. (1999) using the ANKOM Daisy^II^ Incubator system (ANKOM Technologies, Macedon, NY, USA). A key feature of the Daisy^II^ Incubator system was using fiber bags (similar to the bag used for IS assays) to suspend the substrate in the medium so that multiple samples could be assayed in a single vessel (Vogel et al., 1999). The advantages of the IVTD method in the Daisy^II^ Incubator system are a reduction in time and labor, resulting in increased efficiency, standardized vessels with rotation and temperature automation and control, and elimination of individual sample filtration compared to the IVDMD method in individual vials.

The Daisy^II^ Incubator system has been scrutinized for estimating IVTD, especially the disappearance of the various detergent fiber fractions (Tassone et al., 2020). The IVTD method in the Daisy^II^ Incubator system may be less precise than the original Tilley and Terry method (Traxler et al., 1995; Vogel et al., 1999; Wilman and Adesogan, 2000), though the results are similar for forages and relative comparisons that rank values are the same (Holden, 1999; Mabjeesh et al., 2000; Wilman and Adesogan, 2000; Damiran et al., 2008). To address some of the concerns about the IVTD procedure in the Daisy^II^ Incubator, many researchers have sought to address standardizations of the procedure to improve its precision (Tassone et al., 2020). Both sample particle size from grinding and sample amount by weight impact the extent and rate of degradation. In an extreme case, Lentz and Buxton (1992) investigated the effect of highly coarse particle size (8 mm in a shear mill) vs. 1-mm (cyclone mill). These researchers found that the 8-mm particle size yielded greater estimates of potentially degradable cell walls, increased lag time, and decreased rate of disappearance relative to the 1-mm particle size. While ANKOM documentation (ANKOM, 2017) of the IVTD method does not specify a grind size, it is common to use a 2-mm screen in a shear mill for samples undergoing the IVTD assay. Damiran et al. (2008) investigated the effect of shear mill grind size in various IV techniques and found that finely ground (1 mm) substrates tended to overestimate disappearance when compared with more traditional 2-mm particle size substrates. Contrarily, grinding corn silage, grass silage, barley grain, sugar beet pulp, and rapeseed cake at 1-, 2-, 4-, and 8-mm particle sizes in a shear mill did not affect estimating pdNDF degradation parameters using IV or IS methods (Bossen et al., 2008).

In addition to the sample grinding size, sample amount, by weight, has been a topic of interest (Coblentz et al., 2019; Tassone et al., 2020). ANKOM (2017) suggested that a 0.25-g sample weight was sufficient for the IVTD assay but also stated that a 0.5-g sample weight was acceptable. Both Robinson et al. (1999) and Valentine et al. (2019) employed the use of 0.25-g sample amounts, while Trujillo et al. (2010) and Bender et al. (2016) used 0.5-g sample weights for standard 48-h and more than 200-h incubations, respectively. Damiran et al. (2008) reported that DM and NDF digestibility estimates were higher from 0.25-g sample weights than from 0.5-g sample weights. Cattani et al. (2009) compared 0.25- and 0.5-g sample amounts in the Daisy^II^ Incubator system and found that using 0.25-g sample weight resulted in close agreement with results from batch culture incubations and reductions in variance. Coblentz et al. (2019) corroborated these results, finding that 0.25-g sample amounts had better agreement with standard IV procedures across incubation times than 0.5-g sample amounts.

Questions have arisen about the appropriateness of using the ANKOM fiber bags for IV digestibility estimates (Tassone et al., 2020). Wilman and Adesogan (2000) found that the CV was greater for filter bag methods than test tubes, but there was less discrepancy between true and apparent digestibility. The high CV was linked to the escape of soluble particles through the relatively large pores (25 µm) of the ANKOM F57 filter bags (ANKOM Technologies, Macedon, NY). When comparing the ANKOM F57 bags with Dacron bags (more commonly used for IS measurements), Adesogan (2005) found that the ANKOM F57 bags yielded more precise results than the Dacron bags. Valentine et al. (2019) reported that ANKOM F58 fiber bags (8 to 10 µm; ANKOM Technologies) tended to yield inflated undigested NDF (uNDF) values relative to F57, but both were prone to trapping gasses during fermentation that could lead to reduced digestion. More recently, it has been shown that both ANKOM F57 and F58 bags can inhibit fermentation of NDF relative to traditional, beaker-based IV methodologies and yield greater values for fiber following incubation (Schlau et al., 2021).

#### Continuous fermentation

Though continuous culture fermentation technology was known and available since the late 1970s (Hoover et al., 1976; Czerkawski and Breckenridge, 1977), its popularity did not increase until the time following the original symposium in 1991. The first continuous culture system to be proposed was the dual flow system (Hoover et al., 1976). Shortly thereafter, the RUSITEC (RUmen SImulation TEChnique) system was proposed by Czerkawski and Breckenridge (1977), and it is now commercially available through Elementec Scientific Technologies (Maynooth, Co. Kildare, Ireland). The difference between the two systems lies in the outflow models. The RUSITEC system has a single outflow, so digestibility is estimated by suspending samples in the digestion chamber (Stern et al., 1997), whereas the dual flow system has an outflow of liquid and solid fractions (Hoover et al., 1976).

Nocek (1988) touted the viability of the continuous culture system and its advantages over batch culture fermentation in effectively replicating the ruminal environment. This sentiment has continued to the present day. Deitmers et al. (2022) indicated that the automated RUSITEC system reduces the animal-to-animal variation present in the IS technique, decreases human error, and decreases the labor costs of sampling. However, Prevot et al. (1994) stated that the continuous culture system was not a viable option due to its inability to maintain microbial populations similar to the in vivo conditions, though this is also true for batch culture systems and inside IS bags. In a direct comparison of the continuous culture system to ruminally and duodenally fistulated Holstein cows, Mansfield et al. (1995) found that the continuous culture system supported marginally greater concentrations of volatile fatty acids (99.9 vs. 94.9 mM, respectively), greater crude protein digestion (42.9 vs. 38.8%, respectively), lower NDF digestion (47.4 vs. 58.3%, respectively), and similar organic matter digestion (46.8 vs. 47.5%, respectively) than in vivo. The primary critique of the continuous culture system presented by Mansfield et al. (1995) was its difficulty in simulating the digestion dynamics of the various carbohydrate fractions. They suggested this could be due to the relatively lower concentrations of cellulolytic bacteria that were found in the IV system (Mansfield et al., 1995).

Despite the various criticisms of the continuous culture technique for comparison to in vivo conditions, the system continues to see extensive use in the relative comparison of feedstuffs and additives. While not explicitly focused on the dynamics of the cell wall, as was the case at the time of the 1993 symposium, this is especially true of feeding protocols designed to reduce methane output. Since the introduction of the Kyoto Protocol in 1997 and the reports of the Intergovernmental Panel on Climate Change (IPCC), researchers have sought methods in which ruminant animals can be fed to reduce the production of enteric methane (Beauchemin et al., 2022; Tedeschi et al., 2022). Experiments evaluating the inclusion of many different feed additives (Tedeschi et al., 2021), such as exogenous fatty acids (Machmüller et al., 1998; Machmüller, 2006), essential oils (Fraser et al., 2007), biochar (Hansen et al., 2012; Saleem et al., 2018), plant secondary metabolites (Wischer et al., 2013; Dillard et al., 2018; Roca-Fernández et al., 2020), and many other feed additives to assess the reduction in greenhouse gas emissions have employed the use of the continuous culture IV system.

#### Indigestible fiber

Indigestible material at the standardized 48-h end point of fermentation for the IVTD methodology reflects passage kinetics in the rumen; however, it is not an accurate measure of the inherent properties of the cell wall of the substrate, especially for poor-quality forages. Undigested NDF (**uNDF**) is distinguished from iNDF as uNDF is NDF remaining at any given point in time, while the latter is the NDF remaining after a theoretically infinite amount of time under anaerobic fermentation conditions without escaping the rumen (Mertens, 2013; Valentine et al., 2019). Since then, the terms have been incorrectly used interchangeably. The iNDF can hardly be determined on IV systems because it is difficult to maintain a similar hospitable anaerobic environment on IV systems compared to the rumen (e.g., regulation of pH, temperature, and redox while maintaining adequate movement of the substrate, the inflow of substrate and the outflow of fermentation end products, and removal of bacteriocins). Even IS systems have limitations. The most important concept is that iNDF may never be achieved in normal conditions in the rumen as the fraction escapes the rumen; thus, its indigestibility effect might be minimal to the ruminal bacteria for animals under normal feed intake conditions. The need to determine iNDF is more purely mathematical and theoretical than biological. Harper and McNeill (2015) defined iNDF as the fraction of NDF remaining after 288 h of incubation IS. Others consider this to be a representation of the NDF fraction that remains after 240 h IV (Palmonari et al., 2016; Raffrenato et al., 2018). In fact, it is this definition of iNDF that serves as the basis for the Cornell Net Carbohydrate and Protein System (Higgs et al., 2015; Van Amburgh et al., 2015) as iNDF is crucial to models of digestion kinetics and dynamic nutritional modeling (Tedeschi and Fox, 2020a, 2020b).

Developments in the iNDF system have mostly revolved around extending time points for estimating undegradable material or using multiple time points to model disappearance. Bender et al. (2016) found that extending incubation from 120 to 288 h resulted in a decrease in iNDF of 6.6 percentage units, or 17%, and a decrease in the rate of digestion of the pdNDF fraction. Raffrenato et al. (2018) found no difference in uNDF for incubations longer than 240 h when compared to the 240-h sample. Coblentz et al. (2019) observed that the pattern of NDF digestibility moved from quadratic to linear with increasing lengths of IV incubation. There is concern about the viability of inoculum over longer periods of time of IV incubation, though Raffrenato et al. (2018) stated that if the culture is anaerobic with a maintained pH then reinoculation after 120 h is not necessary.

#### Alternate inoculum

The use of rumen inoculum to conduct IV studies can be labor and cost intensive, as well as subject to ethical scrutiny (Mansfield et al., 1995; Castillo and Hernández, 2021). These limitations have inspired several groups to investigate alternative inoculum sources for the estimation of digestibility. In estimating feedstuff digestibility for equine species, it has been accepted that fecal inoculum can stand in place of microbial medium from the cecum (Abdouli and Attia, 2007; Earing et al., 2010; Tassone et al., 2019). Based on this hypothesis, others have investigated the use of ruminant fecal material as an inoculum source representing the rumen. Hughes et al. (2012) determined that bovine feces included at a rate of 450 g/L of bicarbonate buffer was similar to rumen fluid in the estimate of organic matter digestibility coefficients for tropical forages. Chiaravalli et al. (2019), found that fecal inoculum from dairy cows underpredicted NDF disappearance, primarily when incubations were carried out beyond 288 h for the determination of iNDF. It should be noted, however, that the fecal inoculum to buffer ratio was 1:2 in that instance, well under the 45% recommended by Hughes et al. (2012).

In lieu of ruminally fistulated animals, several authors have proposed collecting rumen fluid from recently harvested animals to obtain inoculum (Chaudhry, 2008; Tassone et al., 2014; Sarnataro and Spanghero, 2020; Garcia et al., 2021; Fortina et al., 2022). Chaudhry (2008) was among the first to propose the use of fluid from recently harvested animals and found that DM disappearance was within reasonable limits but cautioned that the pre-harvest feeding regime played a significant role in the viability of the inoculum. Fortina et al. (2022) attempted to standardize the procedure for obtaining inoculum from harvested cattle and found that fluid could be stored for up to 300 min so long as anaerobic conditions and temperature were controlled.

Others have investigated the potential of storing rumen fluid for extended periods of time to meet labor availability and decrease the number of universities and institutions that must maintain fistulated animals. Luchini et al. (1996) evaluated the effect of freezing or lyophilization on the enzymatic activity of rumen inoculum for the IV procedure. In their investigation, freezing mixed ruminal microorganisms followed by pre-incubation provided sufficient proteolytic activity, but they cautioned that this was sufficient only for rank-ordering as it may reduce the rate and extent of digestion relative to fresh, strained rumen fluid (Luchini et al., 1996). Lyophilization was consistently poorer in the preservation of ruminal microbes, and glycerol addition as a cryoprotectant did not improve these results (Luchini et al., 1996). Equivalent results were observed by Garcia et al. (2021) in which both lyophilized and frozen ruminal fluid resulted in decreased IV DM and NDF disappearance relative to the fresh fluid. In agreement, the frozen treatment without a cryoprotectant seemed to offer the most favorable solution for rank-ordering in the absence of fresh rumen fluid (Garcia et al., 2021). Ma et al. (2022) compared IV fermentations with fresh and frozen ruminal fluid and the DM digestion was 4 percentage units less for frozen than fresh inocula; however, when the concentrate: forage ratio was 1:4 the DM digestion was 10 percent units less than a 1:1 ratio. Gas production was reduced when frozen inocula were used through the rank order of 1:4 and 1:1 concentrate: forage remained the same.

### Developments in the in situ technique

Nocek (1988) and Vanzant et al. (1998) addressed the IS technique for determining ruminal digestive kinetics. In these reviews, authors identify the principal factors leading to variation in measurements up to that time, then offer recommendations for standardization of each of these factors. The manuscripts identified several factors, including animal characteristics, substrate characteristics, bag characteristics, temporal characteristics, procedural items, and mathematical calculations (Vanzant et al., 1998).

#### Standardization of methodology

Commercialization has also facilitated progress to standardize IS methodology as currently premade bags with appropriate pore sizes and other supplies are available through Bar Diamond (Parma, ID, USA). However, standardization of the IS technique is more complicated than that of the IV technique due to animal factors. Since 1993, efforts to standardize sources of error in IS methodology have been made; however, there is no universally adopted method. Vanzant et al. (1998) recognized the importance of the digestion kinetic information obtained from IS techniques and the impact of variation internal and external to specific laboratories. Their review summarized the animal, substrate, bag, temporal, methodological, and analytical sources of variation within IS measures and recommended a standardized IS procedure that should improve the precision of digestion kinetic estimates and reduce among and between laboratory variation (Vanzant et al., 1998; Table 1). Two key variation sources that would provide the most improvement in the precision of results identified by Vanzant et al. (1998) were an objective way to determine that postincubation rinsing was complete and quantification of microbial contamination of postincubation sample. The leadership of measures of forage and feed chemical composition is through the Association of Official Analytical Chemists (AOAC, 2023). In situ methodology standardization is outside of their purview; however, other organizations that publish standardized methods could publish recommendations and/or journals could define their expectations for the publication of results. Standard substrates that are included in IV or IS batches or blank bags could be part of method standardization and commercialized products.

**Table 1.** Standardized in situ technique recommended by Vanzant et al. (1998) listed the source of variation in the methodology

Source of variation	Recommendation
Diet	
Type	60% to 70% forage
Feeding level	Maintenance
Feeding frequency	≥2 times/day
Timing of feeding	Not addressed
Bag	
Material	Polyester
Pore size	40 to 60 µm
Sample size: surface area	10 mg/cm^3^
Sample processing	2-mm screen size
Replication	
Number of animals	≥2
Number of days	≥2
Number of bags	≥1
Incubation procedure	
Preincubation	Not necessary
Ruminal position	Ventral rumen
Insertion/removal	Remove simultaneously
Incubation times	To describe curve
Rinsing	
Method	Machine (5 times at 1 min/rinse)
Determine rinsing is complete	Not addressed
Microbial correction	
Include	Yes
Method of quantification	Not addressed
Mathematical model	Simplest available to adequately describe data
Selection	Not addressed
Standard substrate	Yes

Sources of variation not addressed in the original publication were added.

In the 1980s, the evaluation of mechanical rinsing of postincubation bags was first published (Lindberg, 1981; de Boer et al., 1986). Cherney et al. (1990) compared mechanical rinse in a widely available laundry washing machine to hand rinsing and determined that rinsing up to 200 bags in two 2-min cycles was comparable to hand rinsing; however, rinsing for 5-min resulted in an increased particulate loss from the bags. Before this recommendation, based on time, the conventional procedure was to rinse until water was clear, which is subjective to the operator (Balch and Johnson, 1950; de Boer et al., 1986). Coblentz et al. (1997) collected rinse water for analyses of N concentration and the N concentration depended upon the length of time incubated and the number of rinses. The result was a recommendation that 5 rinses for 1-min were necessary to rinse rumen fluid and this procedure is the current recommendation (Coblentz et al., 1997; Vanzant et al., 1998) (Table 1).

#### Indigestible fiber

Much like the advancements in determining indigestible fiber IV, many investigators have found usefulness in determining indigestible residues in the dynamic ruminal environment using the IS technique. Much of the interest and advancement in the determination of indigestible fiber, as iNDF, has been in the length of incubation (Krizsan and Huhtanen, 2013) and the type of bag (Valente et al., 2011; Norris et al., 2019) used for incubation. Krizsan and Huhtanen (2013) evaluated iNDF at 144, 216, and 288 h, finding a decrease in iNDF recovery at each lengthening incubation. Based on these data, the authors suggested that iNDF should be determined at 288 h. Norris et al. (2019), however, found that iNDF continued to decrease through 576 h, indicating that there remained pdNDF beyond the standard 288 h. Valente et al. (2011) found that in a comparison of Ankom F57 fiber bags (25 µm porosity) with nylon or non-woven textiles, iNDF and indigestible acid detergent fiber (**ADF**) were underestimated with the use of traditional nylon bags, likely due to loss of small particles through the relatively large pores. Valente et al. (2011) used these data to suggest that the Ankom F57 or non-woven textile bags were appropriate for the determination of iNDF. Norris et al. (2019) found that F57 bags resulted in a lower recovery of indigestible material than did the newer Ankom F58 bags (10 µm porosity). In addition to the issues with bag type and porosity, these authors also found that quadruplicate samples were insufficient to achieve 90% power in determining iNDF, becoming among the first to address the necessary replication for indigestible fiber determination (Norris et al., 2019).

#### Type of equation for mathematical modeling

Blaxter et al. (1956) proposed that the ruminal digestive process follows a first-order, exponential, non-linear pattern. This was reinforced by Waldo et al. (1972) and resulted in the pioneering publications outlining exponential growth (Ørskov and McDonald, 1979; McDonald, 1981) and decay (Mertens and Loften, 1980) equations of ruminal digestive kinetics, most often measured IS. This exponential model of IS disappearance remained unquestioned throughout the latter 20th century. However, around the time of the 1991 symposium, and especially in the years following, researchers began to propose alternative models of digestive kinetics that may better describe actual ruminal processes. Robinson et al. (1986) postulated that IS digestive kinetics may be described as first-order (exponential), surface area dependent, logistic, or second-order. France et al. (1990) added the option of Gompertz behavior and suggested that disappearance may be from two, competing pools rather than a single, homogenous, potentially ­digestible fraction. Van Milgen et al. (1993) and Van Milgen and Baumont (1995) hypothesized that the behavior of IS disappearance kinetics was better described by microbial mass and activity rates than by substrate properties alone. There have also been suggestions that IS disappearance may follow an age- or gamma-dependent model (Ellis et al., 2005; Tedeschi et al., 2008; Vieira et al., 2008a, 2008b).

#### Parameter estimation

At the time of the 1991 symposium and 1993 publication (Jung et al., 1993), the prevailing wisdom for the estimation of digestive kinetics from IS data was to obtain non-linear parameter estimates from individual experimental units (usually linear combinations of animal and period) then obtain population parameter estimates from arithmetic averaging (Mehrez and Ørskov, 1977; Zanton and Heinrichs, 2009). This was reinforced by Vanzant et al. (1998), who developed a standardized set of recommendations for replication of animal, period, and bags per time point and to fit the simplest available model to describe the data adequately but otherwise offered no insights into mathematical or statistical computations.

However, others have identified flaws in the two-stage fitting of non-linear kinetics data (Tedeschi, 1996). Van Milgen et al. (1993) found that estimates of bias differed between scale parameters (e.g., fractions of the substrate) and kinetic parameters (e.g., rates), whereby kinetic parameters were more subject to increased bias and decreased precision. Zanton and Heinrichs (2009) relied on the postulations of Box (1971) to state that the two-stage fitting is potentially problematic because non-linear regression models are not unbiased, nor are they normally distributed (Hougaard, 1985). Using simulated data, Zanton and Heinrichs (2009) found that non-linear mixed model regression was superior to two-stage fitting in that it accounted for variation in replication, thereby reducing the bias in population parameter estimates. Smith (2017) further postulated that, while non-linear mixed model regression was a superior technique to two-stage fitting, the technique of Zanton and Heinrichs (2009) was flawed because it relied on the a priori assumption of model behavior.

## Future Opportunities for In Vitro and In Situ Techniques

### Commercialization and standardization

Ruminal fluid collected for IV analyses is variable by diet, animal, collection method, storage vessel, temperature, time to the laboratory, and head-space, among many other factors. One way to standardize ruminal fluid is through commercialization. Currently, fresh rumen fluid is sold in 1-L bottles (Bar Diamond, Inc., Parma, ID, USA). Frozen rumen fluid would be more easily stored and transported. There are inherent issues to using mixed microbial populations and enzymes may solve the variability of ruminal fluid or fecal inoculant variability. Enzyme mixtures with standardized digestibility units could be marketed as a ready-to-use products. This may not be ideal because it bypasses the microbial ecology; however, if mixtures of enzymes represented those commonly found in the rumens of animals fed specific diets, then using enzymes is more practical than maintaining mixed microbial population cultures. The biological component does not lend itself to methods of standardization.

Efforts to standardize IV and IS methodology depend on the perceived value of kinetic information from these experiments. Mertens (1993) and Weiss (1994) both concluded, along with other scientists, that accurate and precise estimates of digestibility extent and kinetics were important for dynamic nutritional modeling. The consensus among an international committee would be required, as would a respected organization to publish the committees’ findings. As recognized by Vanzant et al. (1998), recommended methods must be detailed but flexible to allow for the creation of their use to advance scientific knowledge. Including standard procedures in the graduate student training curriculum and support in the peer-review process would also be necessary to promote acceptance among the scientific community. Unfortunately, some sources of error will occur within and between laboratories that cannot be eliminated. This does not imply that attempts to reduce the areas of error within our control should continue to be ignored.

### Estimation of indigestible fraction

Vanzant et al. (1998) suggested that the precision of results from the IS technique would be most improved by developing a standard postincubation rinsing endpoint and quantifying microbial contamination. Improvement in these parts of the IS technique would result in more accurately determining the indigestible fraction. Unfortunately, there has been little to no research in these areas since the recommendation was made in 1998. Evaluation of other objective measures to determine the rinsing endpoint is needed and technological advancements have resulted in inexpensive, smaller, and more portable instrumentation that may be appropriate for this, such as a handheld colorimeter or turbidity meter.

Microbial contamination and correction are important to determine the undegradable fiber fractions more accurately. The NDF and ADF procedures do not remove all microbial contamination from the postincubation substrate (Arroyo and González, 2013). When microbial contamination using ^15^N marker was used, the concentration of degradable NDF of Italian ryegrass (*Lolium multiflorum* Lam.) was overestimated by 3.34%, but only 0.48% for ADF due to the sequential analyses of NDF and ADF (Arroyo and González, 2013). Guevara-González et al. (2015) confirmed that the ruminal effective degradability of NDF was not estimated correctly due to the proportion of residual microbes for oat (*Avena sativa* L.) hay, ryegrass hay, and vegetable-based feeds. The consequence of not correcting for microbial contamination in both studies is the compounding of error through to NDIN and ADIN calculations which had much greater incorrect estimation (Arroyo and González, 2013; Guevara-González et al., 2015). Microbial contamination of postincubation substrate has often been ignored for fiber digestion kinetic calculations, but attention to this issue should be a priority to improve the quality of data resulting from both IV and IS research.

The microbial correction will need to be substrate dependent and is likely not necessary for ADF digestion kinetics. Purine or ^15^N methodologies are not widely available, so alternatives are needed, such as modeling to estimate microbial contamination. Czerkawski and Cheng (1988) described microorganisms in the rumen as within the ruminal fluid, loosely attached to digesta, or firmly attached to digesta. Most cellulolytic activity is associated with attached ruminal microbes and some microbes degrade hemicellulose in order to access cellulose (Van Soest, 1994). Those firmly attached to digesta are not removed with gentle washing (McAllister et al., 1994). Microbial attachment, and thus contamination, is associated with the NDF constituents of cellulose, hemicellulose, and lignin. Modeling to estimate microbial contamination should be based on NDF concentration or forage species and maturity, or alternative marker techniques.

Vinyard and Faciola (2022) concluded that further research on IV methods is needed, especially in regard to combining IV methods to further the use of IV techniques in the field of ruminant nutrition. This inferred an increased use of modeling to fine-tune laboratory methodologies with animal performance, and we presume that modeling to improve the estimation of iNDF could also be fine-tuned. In situ results in less precision than IV methods (Mbwile and Udén, 1991). Mertens (1993) recommended 72-h incubation to measure iNDF, but 48-h has become the most common endpoint as IV has become most commonly conducted in the ANKOM Daisy^II^ Incubator. Norris et al. (2019) determined that pdNDF is present after 288-h IV incubation in forage with condensed tannin present. Combining IV and IS techniques with modeling may improve the precision and accuracy of iNDF estimations, especially for low-quality forages, co-products, or forages with secondary compounds.

### Statistical analyses

Two-stage fitting of IS data to a few degradation kinetic models, to the exclusion of other, potentially better-fit model options, has become normalized within the scientific community. Advances in computing power enable alternatives and in 2017 Smith (2017) developed DIGEST, a SAS macro to automatize the determination of 54 non-linear degradation models identified in the literature and non-linear mixed modeling that was suggested by Zanton and Heinrichs (2009). In essence, the DIGEST procedure fits the data to the best statistical model instead of fitting the data to a selected model. Parameter estimates of the optimum model are then an output of the DIGEST program (Smith, 2017). Data from an IS experiment by Smith et al. (2017) were analyzed with the DIGEST macro and determined that the traditional lag exponential model by Mertens and Loften (1980) ranked 6th, 17th, and 36th as the best fit of DM, NDF, and ADF degradation data, respectively, when the 54 model options were compared (Smith, 2017; Table 2). The best-fit model was not the same for DM, NDF, and ADF degradation kinetics (Table 2), though IS data for DM, NDF, and ADF commonly fit to the same model. Identifying the appropriate model is crucial for low-quality forages, co-products, or other unusual feedstuff since the most used first-order kinetic models are usually not an appropriate fit for the degradation kinetic models of these types of feedstuffs.

**Table 2.** Model selected by DIGEST^1^ model selection macro for “Tifton 85” bermudagrass harvested in June, August, or September combined factorially with 0.0%, 0.25%, or 1.0% BW supplementation with dried distillers’ grains with solubles

Item	Dry matter	Neutral detergent fiber	Acid detergent fiber
Best fit model	Gompertz Model of Ricker (Ricker, 1979)	Generalized Michaelis-Menten Model (Tedeschi et al., 2008)	Von Bertalanffy Model (Ricker, 1979)
Model behavior	Sigmoidal	Sigmoidal	Exponential
Alternative nomenclature for model	Gompertz Growth Curve	Cone model	Pütter’s growth curve no. 2
Akaike weight^2^, %	27.8	26.7	23.8
Ranking of the fit of data to lag exponential model (Mertens and Loften, 1980)	6	17	36

Adapted from Smith et al. (2017) and Smith (2017).

^1^DIGEST is a macro for SAS to fit data from in vitro or in situ experiments to fit 54 non-linear ruminal digestive kinetics models (Smith, 2017).

^2^Akaike weight (Burnham and Anderson, 2004).

### Data science

Advances in computing power facilitate data science solutions to improve the analyses of data collected from IV and IS techniques. Norris et al. (1976) first reported the application of infrared spectroscopy to nutritive value estimations of forages. For many years attempts to develop algorithms to use near-infrared spectroscopy (**NIRS**) to analyze IVTD nondestructively have resulted in mediocre results (Buxton and Mertens, 1991). Improvement in algorithms to estimate IVTD from NIRS results for similar forage species has resulted from improved model fitting techniques (Acosta et al., 2020). Another method to improve algorithm development is through machine learning methods, such as support vector machine (**SVM**) learning algorithms. Baath et al. (2020) applied SVM, which are non-parametric models to identify linear and non-linear relationships between the input vector(s) and output(s), to IVTD estimates by NIRS data to five different warm-season legumes and a global algorithm. Separate calibration and validation data sets were used and SVM resulted in a global model across warm-season legume species of acceptable accuracy to predict most components of nutritive value, except IVTD, compared to partial least squares and Gaussian processes approaches (Baath et al., 2020). The determination coefficient in external validation of the SVM prediction of IVTD was between 0.86 and 0.97 R^2^_*v*_ for three warm-season legume species but below 0.77 *R*^2^_*v*_ for the other two species (Baath et al., 2020). An SVM learning algorithm is supervised; however, unsupervised artificial intelligence data techniques also have a role in this rapidly emerging field.

Artificial neural networks (**ANN**) are a type of artificial intelligence that may be supervised or unsupervised and appropriate for non-linear and complex data sets. Artificial neural networks are designed to mimic the way the human brain functions (Picton, 1994). Nodes, or artificial neurons, are connected processing elements each with layers of input from the user data and the output of the other nodes, hidden layer, and output patterns (Caudill, 1987). Li et al. (2019) conducted a meta-analysis with ANN to predict ruminal pH, ammonia-N, and volatile fatty acid concentrations with more precise but not more accurate results than multiple linear regression. Ebadi et al. (2011) predicted the true digestible amino acid contents from the crude protein, crude fiber, ether extract, ash, and total phenols of sorghum grain for poultry more accurately with ANN than multiple linear regression. The Cornell Net Carbohydrate and Protein System N-fractions and IV ruminal ammonia-N concentration predictions with ANN had greater R^2^ and concordance correlation coefficient and lower root mean square prediction error than alternative modeling methods (Dong et al., 2022). Due to the paucity of information on the application of ANN in ruminant nutrition, especially concerning fiber digestibility, research opportunities exist on the application of ANN to estimation of IV and IS fiber digestion extent and kinetics, iNDF, estimation of in vivo digestibility and intake from these results, and other applications of this data to modeling.

Woli et al. (2020a) used a modeling approach to estimate the daily nutritive value of actively growing bermudagrass [*Cynodon dactylon* (L.) Pers.] dynamically for potential stocker performance. The daily nutritive value of bermudagrass was further used to modify the Weiss et al. (1992) summative equations for TDN by adding a phi (φ) value to account for the daily associations between lignin and cell wall components (Woli et al., 2020b). Thus, the variable φ model used the day of the year to estimate TDN more accurately for warm-season perennial grasses and more accurately predict ADG of stocker cattle. Incorporating the daily nutritive value of bermudagrass and modified TDN, Woli et al. (2022) revealed a modification to the NRC weight gain model to estimate daily gain more accurately for stockers grazing bermudagrass in the southern United States The development of this empirical forage-beef model was applicable to the daily variation in intake and gain throughout the grazing season that accounted for changes in weather, forage maturity, etc. This empirical model is specific to stocker cattle grazing warm-season perennial forages in the southern United States; whereas, mechanistic models are more difficult to interpret and challenging to apply due to a myriad of animal-specific inputs. With the pooling of big data, creative applications of data science, such as the example here, are opportunities; however, the output is only as accurate as the information input into the modeling effort (Tedeschi, 2019, 2022). Digestibility of NDF, as presented in the Weiss et al. (1992) equation is an estimate elicited from lignin encrustation theory that used surface area. It is possible that improving the precision of both IV and IS data to more accurately estimate iNDF and fiber digestion kinetics through the forage growing season would improve the potential of these types of data science applications.

## Conclusions

These techniques are critical to linking nutritive value with forage quality and, thus, to the efficiency of improvements in the field of ruminant nutrition. There has been advancement in the IV and IS techniques and their applications since the International Symposium on Forage Cell Wall Structure and Digestibility in 1991; however, limitations remain. Some limitations to these techniques, such as the variation in ruminal fluid inocula, cannot be managed; whereas other factors, such as determining which model best fits the data, quantification of and correction for microbial contamination, or ­determining when rinsing of postIS bags is complete are within our ability to control and manage. That progress is stagnant in these areas is a tremendous opportunity to improve the precision and accuracy of results from these methods. To approve Animal Use Protocol applications, Institutional Animal Care and Use Committees require that alternatives to animal use were sought. Data science applications can be useful in reducing animal numbers, improving results, and expanding the application of data, but they will not provide new data and information. Mathematic modeling is a powerful and fantastic tool for data and predictive analytics, but it will not sufficiently replace the ubiquitous and critical role of the animal for applications using IV and IS techniques.

## Abbreviations:

ANN: artificial neural network
DM: dry matter
IS: in situ
iNDF: indigestible neutral detergent fiber
IV: in vitro
IVDMD: in vitro dry matter digestibility
IVGP: in vitro gas production
IVTD: in vitro true digestibility
NDF: neutral detergent fiber
NIRS: near-infrared spectroscopy; pdNDF, potentially degradable neutral detergent fiber
SVM: support vector machine
uNDF: undigested neutral detergent fiber
